# From master’s thesis to research publication: a mixed-methods study of medical student publishing and experiences with the publishing process

**DOI:** 10.1186/s12909-024-05060-7

**Published:** 2024-01-20

**Authors:** Maria Björklund, Ramin Massoumi, Bodil Ohlsson

**Affiliations:** 1 Library & ICT, Faculty of Medicine, Lund University, Lund, Sweden; 2 Translational Cancer Research, Department of Laboratory Medicine, Lund University, Medicon Village, Lund, Sweden; 3 Department of Clinical Sciences, Lund University, Malmö, Lund, Sweden; 4 Department of Internal Medicine, Skåne University Hospital, Malmö, Sweden

**Keywords:** Student publishing, Supervisors, Master’s theses, Research publications, Publication process, Student performance, Learning experience

## Abstract

**Background:**

Medical student master’s theses are often carried out as research projects, and some are published as research papers in journals. We investigated the percentage of master’s theses conducted by 5th -year students at the Medical Degree Program at Lund University, Sweden, that subsequently served as the basis for research publications. In addition, we explored both student and supervisor experiences with the publishing process.

**Methods:**

A cohort of four semesters of student data covering the period from 2019 to 2020 (*n* = 446) was searched in PubMed, Embase and the Web of Science to assess whether they had been published as research papers. Surveys were sent to students (*n* = 121) and supervisors (*n* = 77) to explore their experiences with the publishing process.

**Results:**

We found that 33% (149 of 446) of the students in the 2019–2020 cohort subsequently published their theses, and 50% of these students were listed as first authors. Most students published original research. Students (*n* = 21) and supervisors (*n* = 44) reported that the publishing process was time-consuming and that students needed multilevel support from supervisors to achieve successful publication. The publishing process was reported by 79% of the students to have led to additional learning. Most of the papers (126 of 149, 85%) had a clinical or patient-oriented focus.

**Conclusion:**

A high percentage of the student publications in which students are listed as first authors require engagement from both students and supervisors. Supervisors play an essential role in supporting students in a successful publication process. Most of the published papers were either clinical or patient-oriented research.

**Supplementary Information:**

The online version contains supplementary material available at 10.1186/s12909-024-05060-7.

## Background

In medical education, reading research papers, knowing research methods, and performing critical appraisals of research are important for following medical developments and understanding the rationale behind treatment strategies [1, 2]. The approach to student involvement and its application in research-related learning activities seems to vary in form, content and level across medical degree programs. The development of research skills is encouraged for the benefits it brings to the medical profession; e.g., such research is encouraged by the Association for Medical Education in Europe (AMEE) [3]. The involvement of faculty as coauthors with students is one means of engaging students in authentic research projects and can be conceptualized as *research-based learning.* Given that this term has no uniform definition, it can include many activities at different levels, ranging from understanding research content and methods to applying these techniques in the production of research projects or publications [4, 5]. *Research skills* or *research activities* are other commonly mentioned concepts. These concepts encompass the reviewing of research, methodological competencies, reflection, and communication skills along with content knowledge [4, 6]. Research publishing in itself is aimed at reporting and communicating new research findings and the way that an original study was conducted [7]. In medical education, students are trained in reading, understanding, assessing and synthesizing original research papers. For students, writing and publishing a research paper adds an extra dimension to research engagement, implying an active role as an author in engaging in the process all the way to publication. In this scenario, the students not only act as readers or critics but also learn to conduct research projects. The roles and responsibilities of the authors are defined by the International Committee of Medical Journal Editors (ICMJE), which states that authorship implies responsibility and accountability for published work. Authors make substantial contributions to the research, draft and approve the final version to be published, and agree to accountability for all aspects of the work [8]. Subsequently, student-active work is needed to fulfill the author criteria for students, which is addressed through collaboration with their supervisor and research team. Transforming a master’s thesis into a research paper can be an extracurricular activity in which students practice the writing workflow, prepare a manuscript to meet a journal’s format requirements, collect feedback from their coauthors for revision of the text, and finally submit the manuscript, all under supervision. This also includes revising the manuscript after comments from reviewers and editors have been collected, as well as preparing a response to reviewers [7].

### Aim

The main objective of this study is the investigation of the number of 5th -year medical students in the Medical Degree (MD) program at Lund University that succeeded in publishing their master’s theses as research publications. In addition, we explore the experiences of both students and supervisors regarding the publication process and student learning experiences.

## Methods

### Setting

The Faculty of Medicine at Lund University has 2900 full-time students and more than 1000 PhD students [9]. The MD Program in Sweden recently changed from a 5.5 to a 6-year program, and at Lund University, new students are enrolled every semester. Together with medical and clinical knowledge and skills, a curriculum designed to facilitate progressive student learning of research methods and applications, including assessments, is integrated throughout the MD program [10].

### Master’s thesis course, content and structure, learning objectives and assessment

The learning objectives of the master’s thesis course taken in the 5th year of the program are focused on students’ ability to evaluate research papers and understand ethical, juridical, and methodological aspects of the research. Students should be able to create a project plan, run a project under supervision, independently find relevant research and synthesize it into their project background. To help students fulfill these learning objectives, lectures and workshops on research methodology, information retrieval and academic writing are offered.

Prior to their 5th year, the students need to find a supervisor who is available for consultation throughout the course. Supervisors need to hold a PhD in any field applicable to the science and practice of medicine. The thesis is evaluated by an expert assessor with extensive experience in thesis assessment, who then provides feedback to the student’s written thesis and its oral presentation. The evaluation of the written thesis is similar to the research review process of a scientific peer-reviewed journal.

### Data collection

#### Tracking of published student theses

The records of 446 students who completed their master’s theses over the course of four semesters during the 2019–2020 timeframe were reviewed to determine whether their thesis projects had been published as a research paper. If so, the journal and its impact factor, together with the type of publication (original paper/systematic review or other), were documented. To track publications, the databases PubMed (National Library of Medicine), Embase (Elsevier) and Web of Science (Clarivate) were used. The journal impact factor (of 2021) was retrieved from Journal Citation Reports (InCites). The databases were chosen because they were considered to cover the most relevant journals likely to publish these students’ work. The family names of students and supervisors were searched in combination since we considered it less likely that students would publish their work as single authors. Spelling variations were tested for names with special characters or double family names. Ambiguities, either where a student was likely to have made a change in project focus or title or unambiguously connecting authors with a publication or several possible publications was difficult, were followed up on by cross-checking theses records.

#### Surveys to students and supervisors

Surveys were sent to students in the fall 2022 cohort (*n* = 102) and to students in the 2019–2020 cohort, when publications were identified and students provided contact addresses (*n* = 19). The surveys were also sent to supervisors in the fall 2022 cohort (*n* = 77). The survey distributions for the student cohorts and supervisors are described in Table 1.

**Table 1:** Survey distribution to students and supervisors in master’s thesis courses: Overview of cohorts

Survey distribution to students and supervisors in master thesis courses: Overview of cohorts				
Spring 2019	Fall 2019	Spring 2020	Fall 2020	Fall 2022
Survey sent to students in 2019–2020 cohorts who had published their master thesis as a research paper and who also provided a contact address for correspondence in the publication (*n* = 19).				Survey sent to all students in course (*n* = 102), and supervisors (*n* = 77) In this cohort, students were potentially still in the publishing process when receiving the survey.

The survey was designed to collect respondent experiences of the publishing process in regard to student learning, student use of previously acquired research skills, student knowledge of research methods and the level of independence exhibited in student work. The survey questions were answered anonymously and are available in Appendix 1. For practical reasons, the surveys were not sent to the same cohorts as the publication tracking cohorts. Medical students in Sweden graduated after 5.5 years and were difficult to reach for follow-up questions. Nevertheless, we managed to trace certain students who had published their work and provided their personal email addresses for contact purposes. Supervisors are often engaged for many years, as some of them may have served as supervisors in the publication tracking cohort, thus making them easier to contact for follow-up questions.

### Statistical analysis

The statistical analysis of the survey results was performed in SPSS (version 29, 2022). Fisher’s exact test was used for categorical data, and the Mann‒Whitney U test was used for ordinal data. A *P* value ≤ 0.05 was considered to indicate statistical significance.

### Ethical considerations and approval

#### Ethical approval

by the Swedish Ethical Review Authority was waived since the surveys sent to respondents were answered anonymously and the answers could not be traced back to the responders. No sensitive personal data were available for identifying the responders. The responders were informed of the way that how the survey results would be processed and that by answering the survey they were will give informed consent to participate. [Swedish Ethical Review Authority on the Ethical Review act: https://etikprovningsmyndigheten.se/en/what-the-act-says/]

## Results

### Number and type of student publications

The results of our student publication tracking are summarized in Table 2. In total, we tracked 446 student theses, 149 (33%) of which were published as a research paper. The students were the first authors of 50% of the publications, and the most common publication type was original research papers. We also found a few systematic reviews, one narrative review, conference abstracts, a poster and a preprint. The time span of the publication process and the range of impact factors of the journals are illustrated in Table 2. Several students collaborated and published their work in conjunction with fellow students as part of a research group. We counted the individual student contributions to publication; if two students in the same cohort had a joint publication, we counted both students as publishers.

**Table 2:** Number and type of student publications

Year/cohort/ number of students	Number of publications	Student as first author	Original papers	Systematic reviews	Narrative reviews	Conference abstracts	Posters	Pre prints	Journal impact factor	Time to publication
2019 Spring *N* = 97	*n* = 33 (34%)	18 (55%)	25	3	1	3	0	1	1.023–15.483	5–42 months
2019 Fall *N* = 116	*n* = 42 (35%)	17 (40%)	37	2	0	3	0	0	1.023–16.051	2–26 months
2020 Spring *N* = 115	*n* = 39 (34%)	23 (59%)	35	2	0	2	0	0	1.023–38.637	4–32 months
2020 Fall *N* = 118	*n* = 35 (30%)	16 (46%)	33	1	0	0	1	0	1.565–15.441	3–24 months
**Total ** *N* = 446	***N*** ** = 149 (33%)**	**74 ** **(50%)**	**130**	**8**	**1**	**8**	**1**	**1**	**1.023–38.637**	**2–42 months**

Most of the 149 published papers (85%) had a clinical or patient-oriented focus, for instance, diagnostics, screening, clinical management, therapy, follow-up and prognostics, complications, lifestyle, risk factors/risk management in health care, or mental health. Some of the study designs applied included randomized controlled trials, clinical trials, observational studies, multicenter studies, and registry studies.

### Survey results of student and supervisor experiences with publishing

Forty-four (57%) supervisors and 21 (17%) students responded to the survey, but not all respondents answered all questions. Of the total number of student respondents, 9 (43%) had published their theses as research publications, while 12 (57%) were in the process of publishing. All the responding supervisors reported that they had worked with students who had or were about to publish. In general, supervisors provided us with extended comments in their responses, which contributed to capturing more in-depth perspectives in regard to their experiences.

### Learning from the publishing process

Sixteen (84%) of the 19 students responding on the question indicated that they applied their previous learning of research methodology to their publishing endeavors, whereas 3 (16%) reported that they did not utilize their previous learning in this regard. Two students expressed that their previous curricular activities in research methodology served as a suitable foundation for their thesis. 27 (64%) of the 42 supervisors responding on the question reported that students applied skills and knowledge from previous learning activities and that many took a scientific approach to the work. There were also individual variations in the levels of student preparedness. However, as 15 (36%) of the supervisors noted, the publishing process is new to students, and they often need considerable guidance. Moreover, publishing does not occur without any previous knowledge or experience with research methodology. Most of the students (79%) reported that the publishing process led to additional learning, in contrast to 21% who reported that they did not incur any extra learning. The time and effort required for publishing, including generating more advanced statistics, adapting the thesis to a journal paper format, and responding to peer review and communication skills, were mentioned as specific new learning experiences.

A majority of the supervisors (95%) reported that students who published also gained additional experience from the research methodology in the sense of deepening their knowledge of the medical topic. The difference between the peer-review process and the examination process for theses was reported as another aspect of learning. The supervisors acknowledged the positive learning effects for students who authored published research papers in various aspects of managing the project, preparing for submission, and adhering to deadlines. In contrast, a few supervisors noted that not all students were able to perform these tasks independently and that some required substantial assistance. Supervisors also expressed that some students lacked patience; e.g., some students expressed impatience in working with adaptations of the thesis and responding to reviewer comments.

### Support needs in the publishing process

**Fig. 1:**
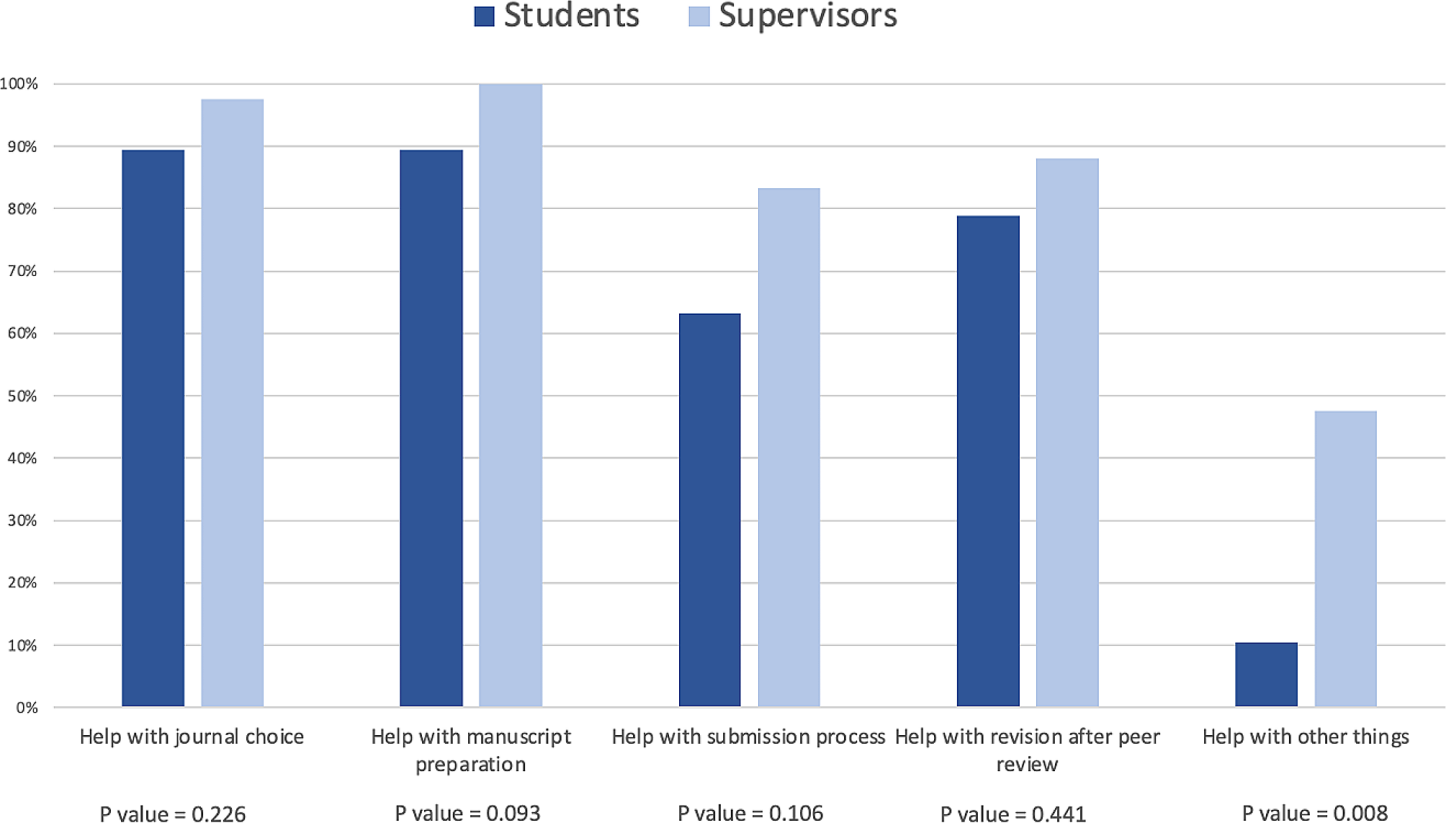
Student support needs in the publishing process: Comparison of student (n = 19) and supervisor (n = 42) experiences. Fisher’s exact test was used. A P value ≤ 0.05 was considered to indicate statistical significance

All the students emphasized the significant need for assistance throughout the publishing process, but they provided few detailed comments in their responses. Supervisors expressed a similar need to support the students; see the comparison in Fig. 1.

Supervisors reported that they helped students connect with the other coauthors, which was beneficial for student learning. In the publishing process, students are required to adapt their theses to specific journal requirements and write more concise and clear texts, which, according to some supervisors, took more time than students thought it would. Proofreading and obtaining help in formatting figures and tables were also mentioned as very important factors. Some supervisors reported that additional material or statistical analysis as well as methodological considerations were sometimes needed to succeed in publishing.

The supervisors also commented that the quality levels of theses guided their decision to pursue publication and engage with students as coauthors. Some students also required help with communicating their publication at conferences or meetings, according to supervisors.

### Level of student independence in the publishing process

Students generally reported having some or a low level of independence in the publishing process. A few reported higher levels of independence, either in writing the manuscript and/or in data analysis.

**Fig. 2:**
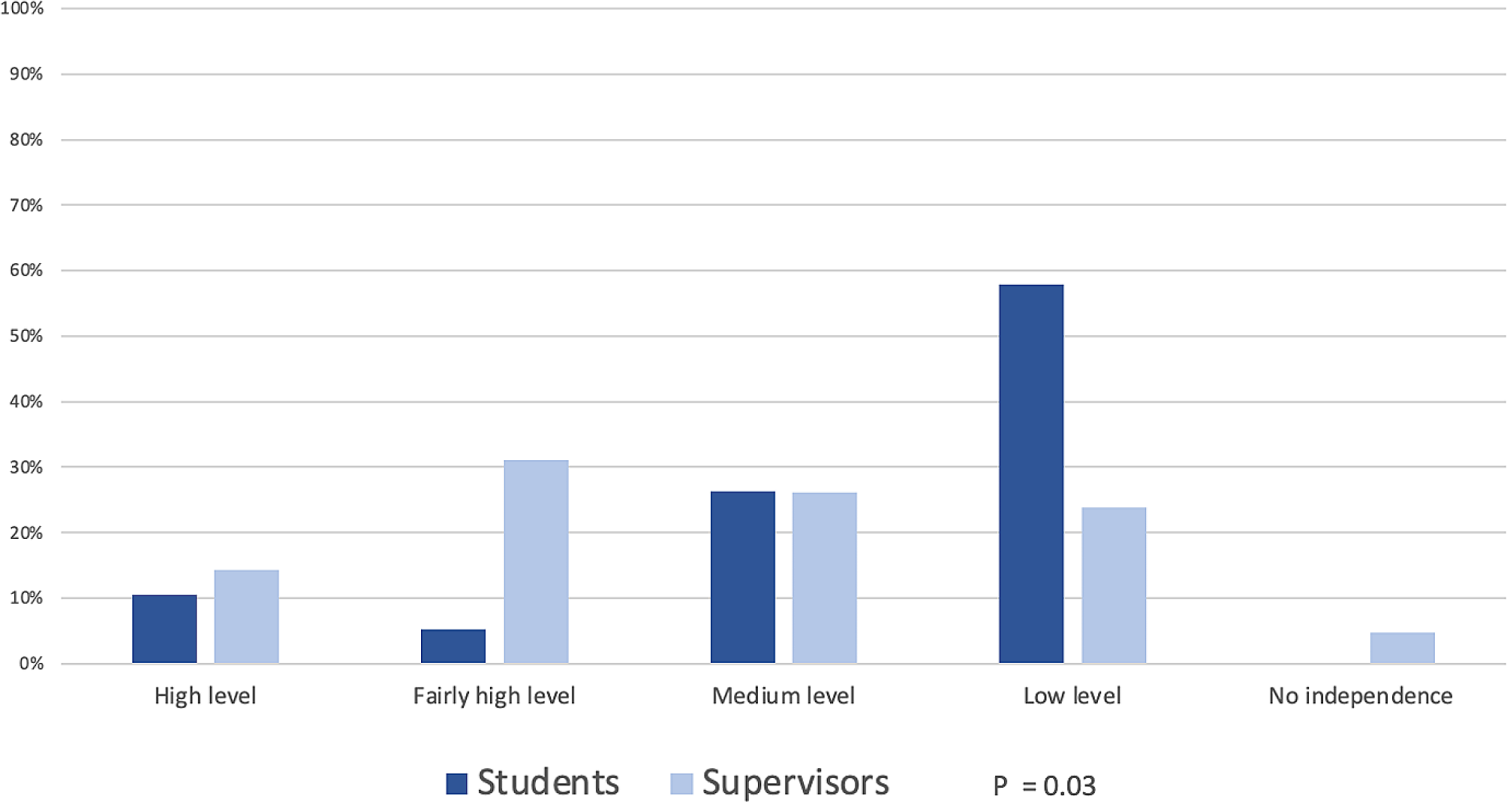
Level of student independence in the publishing process: Comparison of student and supervisor experiences Students (*n* = 19) and supervisors (*n* = 42) rated the perceived levels of student independence on a five-point scale. Mann‒Whitney U test was used. A *P* value ≤ 0.05 was considered to indicate statistical significance

Supervisors reported slightly higher levels of independence on the part of students and commented that the level of independence varied across students. A comparison of perceived independence is presented in Fig. 2.

## Discussion

In this study, we investigated a cohort of medical degree students over four semesters ranging from 2019 to 2020 to assess whether their master’s theses had been published as research papers. We found that 33% of the articles had been published. Different methods have been applied in previous research on student submissions, publishing rates and student authorship; Skovgard et al. [11] and Griffin et al. [12] followed up on student cohorts, while Kan et al. [13] and Svider et al. [14] investigated specific journals for student publications. The extent to which and how research activities are integrated into the curriculum may also vary across educational and geographical contexts. Most of the studies were from the United States, followed by the United Kingdom and Australia, as reported by Carberry et al. [15]. This makes comparisons difficult because of the differences in educational contexts and methods of publication tracking among these contexts. The share of student authorship varies: Skovgard et al. [11] studied a cohort of Danish students where 52% managed to publish at least one paper, and Griffin et al. [12] reported a UK student cohort where 14% (72 of 515) of the authors submitted research articles for publication. Kan et al. [13] and Svider et al. [14] tracked student publications in journals and revealed student authorship percentages of 12–19% and 19–37%, respectively. Amgad et al. [16] estimated student publishing rates to be 25–30% in their meta-analysis. Our results of 33%, show a higher rate of student publication than previous studies showed. In this group, 50% of the students were first authors, which is a high proportion in comparison to the findings of previous studies, including those of Amgad et al. [16], where 13% of the students were first authors, while Kan et al. [13] and Skovgard et al. [11] reported 17–25% and 43%, respectively.

The experiences reported by students and researchers showed that the publishing process can be beneficial for student learning. There were differences between student and supervisor experiences regarding student levels of independence, where some students reported their estimated level of independence to be lower than that reported by their supervisor. The reports of the students regarding support needs were similar to those of their supervisors. Supervisors emphasized that students required substantial assistance to a greater extent than the students themselves did, and such assistance seemed to be vital to a successful publication process. This result is also in line with the findings of previous studies in which students described having an engaged supervisor or mentor as the most helpful factor, followed by the support of the research team, course leaders and peers [12, 17, 18]. This heavy reliance of the student on the supervisor was also reported by Althubaiti et al. [19]. Our results reveal that students valued training in efficiently working with a team, which was also reported as a positive outcome in previous research [14, 20]. Maher et al. [21] described a faculty culture in which the publishing process is regarded as a socialization process leading to academic authorship, team collaboration and competent writing as an important factor affecting student-faculty publishing.

Our results reveal that most of the publications of our student sample seems to be clinically or patient-oriented. In previous research, the focus (such as basic or clinical science) and form (reviews or original papers) of student publications seemed to vary depending on the educational context. Stockfelt et al. [6] reported that 45% of students perform basic science or laboratory projects, approximately one-third of the students engage in clinical research, and the remaining students pursue a combination of research activities. Wickramasinghe et al. [22] reported that reviews, followed by original studies, are the most common form of student publication. Amgad et al. [16] reported that most students publish original research papers, and whether these are in basic or clinical science varies, but the majority of them are on the clinical side, which is well aligned with our results.

Even though many of our students go on to publish, we do not believe that requiring such publication as part of the program would be feasible or result in positive outcomes. Voluntary publishing has also been discussed in previous studies, which have emphasized its importance for student motivation and for developing a sound future research culture [15, 18, 23]. Helping students become motivated to engage in research by progressively working with research competencies that have been integrated into a curriculum that culminates in a master’s thesis is likely a better strategy [10]. This longitudinal approach can have positive effects on student publishing, as suggested by Mullan et al. [24], even if such a curriculum has yet to be evaluated.

### Limitations

In our data collection, we aimed for a rigorous approach in determining whether a student thesis had been published as a research paper. However, it is possible that some of the student theses were published in journals that were not indexed by the databases chosen for publication tracking. Another limitation is that students in the fall 2020 cohort may still be in the process of publishing and hence may not be captured in our data. With these limitations in mind, we believe our investigation has resulted in a general and fairly accurate overview of the number of theses that are published as research papers.

We sent the survey to a partly different cohort than the one used for publication tracking. As the survey responses were anonymous, it was not possible to distinguish the different cohorts within the survey results. It is possible that students who have already published have had different learning experiences than students who are still in the publishing process. However, the experiences expressed by students, the impact on learning, and the role of supervisors were found to be more closely associated with the publishing process itself than contingent on the specific cohort to which a student or supervisor belonged. The surveys allowed for detailed comments in the responses, and the supervisors used that option more than students. Due to the low response rate of students, the low rate of student comments, and the lack of representativeness of the results, further investigations into student learning experiences during the publishing process are needed.

## Conclusion

In this study, we investigated the percentage of medical students in the 2019–2020 cohort who subsequently published their master’s theses as research publications. We found a 33% publication rate, and students were listed as the first author in 50% of the publications. The publishing process demands a significant amount of time, and students need to be aware of the additional time required in addition to their master’s thesis work.

Engaged supervisors were found to be essential for a successful publication process, as they provided students with the necessary support in preparing their manuscripts. Student publishing can constitute an additional learning activity in student research projects, provided that such publishing is voluntary and builds on students’ intrinsic motivation to perform research.

### Electronic supplementary material

Below is the link to the electronic supplementary material.

Supplementary Material 1

## Data Availability

The datasets used and/or analyzed during the current study are available from the corresponding author upon reasonable request.

## Abbreviations

AMEE: The Association for Medical Education in Europe
ICMJE: International Committee of Medical Journal Editors
MD: Medical Degree
