# Supplementary material for: From master’s thesis to research publication: a mixed-methods study of medical student publishing and experiences with the publishing process
Source: BMC Med Educ. 2024 Jan 20;24:75. doi: 10.1186/s12909-024-05060-7 (PMC10800057; doi:10.1186/s12909-024-05060-7)
Supplement: Supplementary file 1 — Supplementary Material 1 [file 12909_2024_5060_MOESM1_ESM.docx]

# Survey questions to students [Translated from Swedish]

**Have you published your master thesis as a research publication in a journal?**

Yes/No

Comments

**Are you planning to** **published your master thesis as a research publication in a journal?**

Yes/No

Comments

**In your experience, can you apply what you have learned from previous curricular activities on research methodology, in your publication process?**

Yes/No

Comments

**In your experience, does the publishing process contribute to learning something extra, apart from the learning objectives for the master thesis course? Please specify.**

Yes/No

Comments

**What support did you receive from your supervisor in the publishing process? Multiple responses are possible.**

Help with journal choice

Help with manuscript preparation

Help with submission process

Help with revision after peer review

Help with other things

Comments

**What level of independency do you perceive you have had in the publishing process?**

Rating scale 1-5, 1=no independency 5= high level of independency

Comments

**If you have published, has your publication influenced your role as physician, career options or continued research engagement?**

Yes/No

Comments

**Have you registered for a PhD program after your master thesis course?**

Yes/No

Comments

**If you have additional comments, feel free to share them here**

Comments

# Survey questions to supervisors [Translated from Swedish]

**Have you supervised students who published, or are about to publish their master thesis as a research publication in a journal?**

Yes/No

Comments

**In your experience, do students apply what they have learned from previous curricular activities on research methodology, in the publication process?**

Yes/No

Comments

**In your experience, do the students learn something extra from the publishing process, apart from the learning objectives for the master thesis course? Please specify.**

Yes/No

Comments

**What support have you offered students in the publishing process? Multiple responses are possible.**

Help with journal choice

Help with manuscript preparation

Help with submission process

Help with revision after peer review

Help with other things

Comments

**What level of independency do you perceive students had in the publishing process?**

Rating scale 1-5, 1=no independency 5= high level of independency

Comments

**Have students registered for a PhD program after the master thesis course?**

Yes/No

Comments

**If you have additional comments, feel free to share them here**

Comments
